# Supporting Risk Assessment: Accounting for Indirect Risk to Ecosystem Components

**DOI:** 10.1371/journal.pone.0162932

**Published:** 2016-09-15

**Authors:** Cathryn Clarke Murray, Megan E. Mach, Rebecca G. Martone, Gerald G. Singh, Miriam O, Kai M. A. Chan

**Affiliations:** 1 Institute for Resources, Environment and Sustainability, University of British Columbia, Vancouver, British Columbia, Canada; 2 North Pacific Marine Science Organization (PICES), Sidney, British Columbia, Canada; 3 Center for Ocean Solutions, Monterey, California, United States of America; 4 Fisheries & Oceans Canada, Institute for Ocean Sciences, Sidney, British Columbia, Canada; Northwest Fisheries Science Center, UNITED STATES

## Abstract

The multi-scalar complexity of social-ecological systems makes it challenging to quantify impacts from human activities on ecosystems, inspiring risk-based approaches to assessments of potential effects of human activities on valued ecosystem components. Risk assessments do not commonly include the risk from indirect effects as mediated via habitat and prey. In this case study from British Columbia, Canada, we illustrate how such “indirect risks” can be incorporated into risk assessments for seventeen ecosystem components. We ask whether (i) the addition of indirect risk changes the at-risk ranking of the seventeen ecosystem components and if (ii) risk scores correlate with trophic prey and habitat linkages in the food web. Even with conservative assumptions about the transfer of impacts or risks from prey species and habitats, the addition of indirect risks in the cumulative risk score changes the ranking of priorities for management. In particular, resident orca, Steller sea lion, and Pacific herring all increase in relative risk, more closely aligning these species with their “at-risk status” designations. Risk assessments are not a replacement for impact assessments, but—by considering the potential for indirect risks as we demonstrate here—they offer a crucial complementary perspective for the management of ecosystems and the organisms within.

## Introduction

Most of the world’s ecosystems and the services they supply are threatened as a result of a wide-array of human activities [1–2]. Many overlapping human activities have diffuse impacts with time lags that vary over small temporal and spatial scales, such that quantitatively demonstrating an impact is time-consuming, expensive, and often impossible without more data than are practicable with available resources. Despite these challenges, decision makers still need methods to evaluate the distribution and intensity of land, coastal and ocean-based human activities, and the resulting impacts across species, habitats and ecosystems. Risk assessment is one approach decision-makers use for evaluating the risk to ecosystem components resulting from the suite of multiple human activities and their associated pressures by specifically including uncertainty with impact [3–6], making risk-based approaches valuable and unique for prioritizing management interventions. In particular, risk assessment can be applied rapidly, integrating the best-available science in management of natural resources.

Risk assessments have been implemented across a wide variety of ecosystems [3, 7–10]. There are many ecological interactions that shape ecosystem dynamics and the delivery of ecosystem services that may be disrupted by external pressures, including stressors derived from human activities. Fisheries and Oceans Canada recently developed an ecological risk assessment framework to evaluate the cumulative impacts of multiple stressors on multiple ecological components [11]. The framework has been trialed as a pilot project [12] and applied to marine protected areas to support ecosystem-based management [13]. As it currently stands, the framework focuses only on direct impacts and ignores connections among ecosystem components and the potential for disruption of these ecosystem dynamics. Risk assessment methods of this type may inappropriately ascribe management priority and neglect ecosystem components that may be greatly impacted from human pressures [14]. Indeed, indirect impacts to species by changes in key habitats, predators or prey may be the primary way that impacts manifest [15–17].

Ecological theory predicts several pathways by which ecosystem components would be subject to adverse indirect effects. One primary mechanism by which species experience indirect threats from human activities is through the availability of prey and appropriate habitat. For example, species at the top of the food chain or at higher trophic levels may be at higher risk than predicted from direct impacts alone because these species are often linked to several others in the food web through multiple trophic levels. Indirect effects may magnify impacts with each additional step in the food web. Chemical contamination at higher levels of biological organization is one example of increasing risk mediated by indirect effects through increasing trophic levels [18–20]. Species with highly specialized prey or habitat requirements also may be at higher risk because they do not have the ability to switch resources when conditions change and resource availability or abundance is low. For example, highly specialized sharks are more extinction-prone than their generalist congeners [21]. Key species that form habitats or have high per-population or high per-individual interaction strengths are likely to have significant influence on ecosystem dynamics [22–23] and thus understanding the cascading effects that follow direct impacts to these key nodes may be particularly critical to evaluate. Prey species and habitat-forming species are two types of species that often influence these dynamics in marine ecosystems.

Here we detail and apply a novel method for considering both direct impacts and those mediated through habitat and prey species, to evaluate the potential cumulative impacts from human activities on ecosystem components. We evaluate the relative contribution of direct and indirect risk to species and habitats from a suite of human activities across a range of trophic levels in a northern temperate marine ecosystem, adapting methodology from an existing ecosystem risk assessment framework that considers only direct effects [11]. We then compare how much additional risk is accounted for when indirect effects are included in a risk assessment. Furthermore, we statistically test whether species in higher trophic levels are more likely to be impacted through indirect effects on their prey than species lower in food webs in the modelled system.

## Materials and Methods

### Pacific North coast case study

The Pacific North coast of British Columbia, Canada is host to a wealth of resources important for ecological, economic and cultural reasons, many of which are unique to the region; for example glass sponge reefs, globally significant seabird populations, salmon, eulachon, and resident orca. A broad range of human activities occur in this region, as reviewed in [24]. Sea-based activities include fishing, aquaculture, tourism, utility and transportation. Coastal activities also influence the marine and estuarine resources in this region, including human settlement, ports and marinas, and log storage and handling. Land-based activities occurring in the watersheds are connected to coastal marine systems through freshwater runoff and include forestry, agriculture, mining and pulp and paper mills. The region is also subject to impacts from long-range and global stressors such as climate change, pollutants and debris (Table 1). Activities that include vessel use additionally include the stressors associated with either small or large vessel use in their cumulative risk.

**Table 1:** Sector, human activities and associated stressors included in the risk assessment.

Sector	Activity	Stressor		Sector	Activity	Stressor
Sea				Fisheries		
	*Finfish Aquaculture*				*Dive Fisheries*	
		Acoustic				Direct Capture
		Contaminants				Habitat Disturbance
		Fish Escapement				Small Vessel Use*
		Nutrient Input			*Gillnet Fisheries*	
		Predatory Control				Bycatch
		Small Vessel Use*				Direct Capture
	*Large Vessel Use*					Small Vessel Use*
		Acoustic			*Hook and Line*	
		Contaminants				Bycatch
		Invasive Species				Direct Capture
		Nutrient Input				Small Vessel Use*
		Oil Spill			*Recreational Fishing*	
		Vessel Strikes				Bycatch
	*Log Handling*					Direct Capture
		Contaminants				Small Vessel Use*
		Debris			*Seine Fisheries*	
		Habitat Disturbance				Bycatch
		Small Vessel Use*				Direct Capture
	*Marine Tourism*					Small Vessel Use*
		Disruption of Wildlife			*Trap Fisheries*	
		Habitat Disturbance				Bycatch
		Small Vessel Use*				Direct Capture
	*Ports*, *Marinas*, *Harbours*					Small Vessel Use*
		Change in Water Flow			*Trawling*	
		Contaminants				Bycatch
		Habitat Disturbance				Direct Capture
		Large Vessel Use				Habitat Disturbance
		Nutrient Input				Large Vessel Use*
		Small Vessel Use*				Sediment Suspension
	*Shellfish Aquaculture*				*Trolling*	
		Invasive Species				Bycatch
		Shading				Direct Capture
		Small Vessel Use*				Small Vessel Use*
	*Small Vessel Use*				*Hand Digging*	
			Contaminants		Direct Capture	
		Incidental Mortality		**Land**		
		Invasive Species			*Human Settlement*	
		Nutrient Input				Contaminants
		Oil Spill				Debris
						Nutrient Input
	*Water Diversions*					Sedimentation
		Barrier to fish passage			*Land-based Activities*	
	* *	Change in Water Flow				Contaminants
						Nutrient Input
						Sedimentation
						Temperature change
				**Long Term**		
					*Climate Change*	
						Ocean Acidification
						Sea level rise
						Temperature change
					*Long Range Contamination*	
						Marine Debris
					* *	Persistent Organic Pollutants

* denotes sub-activities (Small Vessel Use and Large Vessel Use) that include the stressors from each of those activities.

### Assessing Risk

We utilized a risk assessment framework for evaluating the risk to species and species groups (hereafter called ecosystem components, Table 2) from stressors associated with various human activities [11]. Risk was estimated as the product of the *Exposure* of a population to a specific human stressor and the *Consequence* or the sensitivity of that population to that same stressor. Risk scoring of each ecosystem component was done considering the species or habitat’s entire life history (rather than examining different life history stages separately). More details of the risk framework can be found in [11] and S1 File.

**Table 2:** Ecosystem components assessed in the qualitative risk assessment. Trophic groups with * are also included in the food web as prey species.

Trophic Group	Ecosystem component	Scientific name
Phytoplankton*	Phytoplankton	
Zooplankton*	Zooplankton	
Habitat-forming macrophytes	Kelp	
	Seagrasses	*Zostera* spp.
Habitat-forming invertebrates	Cold-water corals	
	Sponges	Hexactinellid, cloud, etc
Low mobility invertebrates*	Geoduck clam	*Panopea abrupta*
Mobile benthic invertebrates*	Dungeness crab	*Cancer magister*
Mobile pelagic invertebrates*	Prawn	*Pandalus platyceros*
Anadromous fishes*	Salmon	*Onchorhynchus* spp.
Elasmobranchs	Spiny dogfish	*Squalus acanthias*
Benthic fishes*	Lingcod	*Ophiodon elongatus*
Forage fishes*	Pacific herring	*Clupea pallasi*
Baleen whales	Humpback whale	*Eumetopias jubatus*
Toothed whales	Resident Orca	*Orcinus orca*
Pinnipeds	Steller sea lion	*Eumetopias jubatus*
Seabirds	Cassin’s Auklet	*Ptychoramphus aleuticus*

### Qualitative Risk Assessment

The relative *Risk*_ij_ to an ecosystem component was calculated by multiplying its exposure to a stressor by the consequence of that exposure, or

$${Risk}_{ij}={Exposure}_{i}\times {Consequence}_{ij}^{2}$$ Equation 1

where *Risk*_*ij*_ to ecosystem component *j* by stressor *i* is the product of the *Exposure*_*i*_ of stressor *i* and the *Consequence*_*ij*_ to ecosystem component *j* at being exposed to stressor *i*; where ecosystem component *j* is one of the pilot ecosystem components selected for this analysis (Table 2) and stressor *i* is a stressor produced by one of the sea or land-based activities (Table 1). Qualitative scoring of the risk variables used the same scoring methodology defined by [11] and scored by [12] (Table A in S1 File). *Exposure*_*ij*_ of ecosystem component *j* to stressor *i* was the product of three variables (scored between 0–3; score maximum = 36): *Temporal Scale*_*i*_ *(TS)*, *Spatial Scale*_*i*_ *(SS)*, and *Load*_*i*_ *(L)*:

$${Exposure}_{ij}={TS}_{ij}\times {SS}_{ij}\times L_{ij}$$ Equation 2

Consequence was scored from 1 to 6 for the ecosystem component at the scale of individual stressors. *Consequence*_*ij*_ was squared to make the scale of the score comparable to *Exposure*_*i*_ (such that both have a maximum score of 36). This way, exposure and consequence effectively vary along the same scale even though we broke exposure into three subcomponents that we could score separately and had only one overall score for consequence. An example of the risk calculation (Eq 1) is presented in S1 File. The original risk scores for individual ecosystem component-stressor combinations were assigned after review of the available literature and consultation with experts. The scores are available as S3 File and scoring justifications in ([12] Appendix 4).

### Incorporating Uncertainty into Risk Scores

For each of the four risk variables an uncertainty term was assigned (Table B in S1 File), and Monte Carlo simulation was used to incorporate this uncertainty explicitly into the calculation of risk. Each risk variable score (*Temporal Scale*, *Spatial Scale*, *Load* and *Consequence*) was assigned as the mean of a normal distribution with standard deviation as a function of the level of uncertainty assigned; higher uncertainty had higher standard deviation. The distribution was bounded by the minimum and maximum scores for each risk variable so that the scores estimated to be higher or lower than the variable’s scale were assigned as the minimum and/or maximum (*e*.*g*., a Load score higher than 3 would be scored as 3). The score of each risk variable was then randomly sampled from this distribution with 1000 replicates so that each risk variable was therefore an array with 1000 entries. The final risk score for each ecosystem component-stressor relationship was a product of the four arrays representing the risk variables (*Risk* = *SS* x *TS* x *L* x *C*^2^), where the first score generated from each variable array is multiplied across all four risk variables, followed by the second, and so on for all 1000 replicates and resulting in a final risk array of 1000 scores. The median and 10% and 90% quantiles from this final array of the overall risk to each ecosystem component-stressor relationship was reported. Quantiles were used instead of standard deviation or standard error because the resulting distribution of risk scores was non-normal.

### Cumulative Risk

Cumulative risk to each ecosystem component was calculated by summing the total risk score produced for each ecosystem component across all its stressors, within each iteration of the Monte Carlo simulation. The statistical platform R was used to generate and run the code for the uncertainty scoring (R Development Core Team 2008); code available in S2 File. Direct cumulative risk *CRisk* to the ecosystem component is calculated by summing risk across all stressors so that

$${CRisk}_{j}=\sum_{i}{Risk}_{ij}$$ Equation 3

### Direct and Indirect Risk

**Fig 1:**
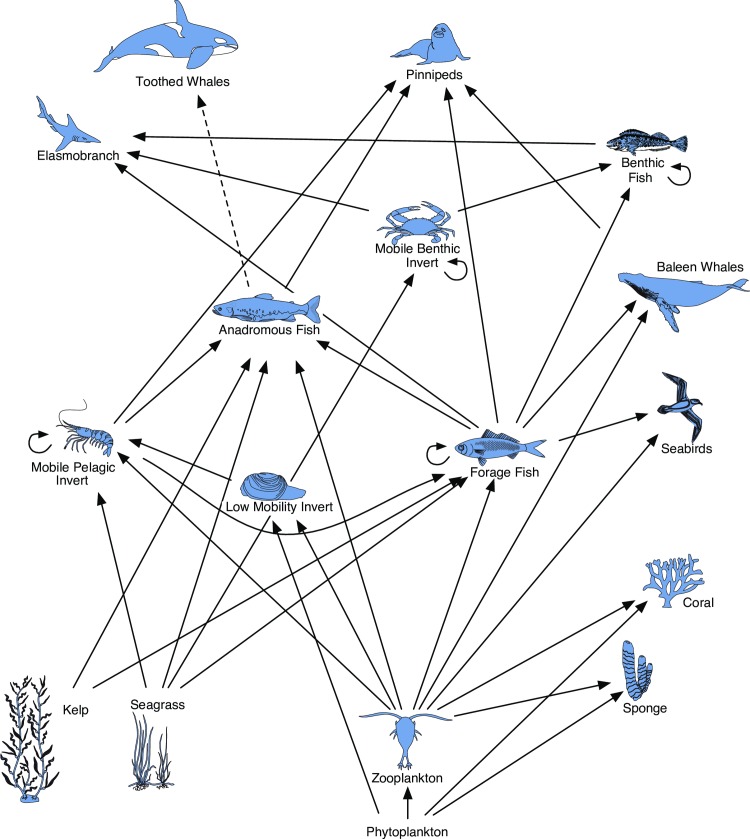
Risk pathway (food web and biogenic habitat) for all species considered. Dashed line = Obligate relationship (100% risk transfer).

In order to incorporate indirect risk through food web and habitat relationships we developed a series of linkage pathways between the ecosystem components and the prey and habitat ecosystem components on which they each depend (Fig 1). The linkage pathways were developed specifically for the study region using literature review (e.g. [25]) and expert consultation. The modeled relationships between species are known to vary based on location, season, year, etc and therefore the pathways represent generalized prey and habitat linkages. Including indirect risks, the *comprehensive* cumulative risk to each ecosystem component *j* is calculated as

$${CCRisk}_{j}={CRisk}_{j}+\sum_{s}{p\;CCRisk}_{s}$$ Equation 4

which includes 100% of its direct cumulative risk (*CRisk*_*j*_), plus a proportion (*p)* of the risk to each of its supporting species (*s)*, including prey species or biogenic habitat (if applicable). We applied a conservative, well-accepted energy transfer relationship of 10% [26] to reflect relationships between species in the network. Because calculating substitutability of prey items and incomplete spatial overlap between predator and prey is extremely data intensive and uncertain, even for a single species, we have made the assumption that, in the absence of complete dependency, the risk is diminished an order of magnitude in the transfer, following the 10% energy transfer principle. If the prey or habitat relationship is obligate (the species is entirely dependent on the prey or habitat species), 100% of the prey or habitat risk is added. For example, we applied this obligate relationship in the case of resident fish-eating orca and their obligate prey species salmon. In case the risk to a known prey species was not scored during the case study, the risk to the trophic groups was estimated by adding 10% of the risk to the ecosystem component representative (e.g. for any species consuming a pelagic forage fish other than Pacific herring, we added to its cumulative risk 10% of the cumulative risk to Pacific herring; all representatives for trophic groups are described in Table 2). As a full example, comprehensive cumulative risk to the ecosystem component Steller sea lion is estimated by the direct risk to Steller sea lion plus 10% of the risk to its prey items (relationships demonstrated in Fig 1): anadromous fish (salmon), mobile pelagic invertebrates (prawn), forage fish (Pacific herring), benthic fish (lingcod). In turn, the comprehensive cumulative risk to salmon was calculated using its linkage framework, including the biogenic habitats kelp and eelgrass.

### Analyses

Median and error (10% and 90% percentiles) of direct, indirect and cumulative risk were calculated and compared across ecosystem components. We tested for a relationship between the median direct and indirect risk scores for each ecosystem component using the nonparametric Spearman’s rank correlation test. Because the indirect risk framework depends on the structure and number of linkages in the food web, we used the Spearman rank correlation test again to compare the relationship between number of prey and habitat linkages and indirect risk. Because results of this test were strongly driven by an outlier, resident orca, which also differs from the other ecosystem components as being the only one with an obligate feeding relationship (with salmon), the test was repeated without this species.

## Results

**Fig 2:**
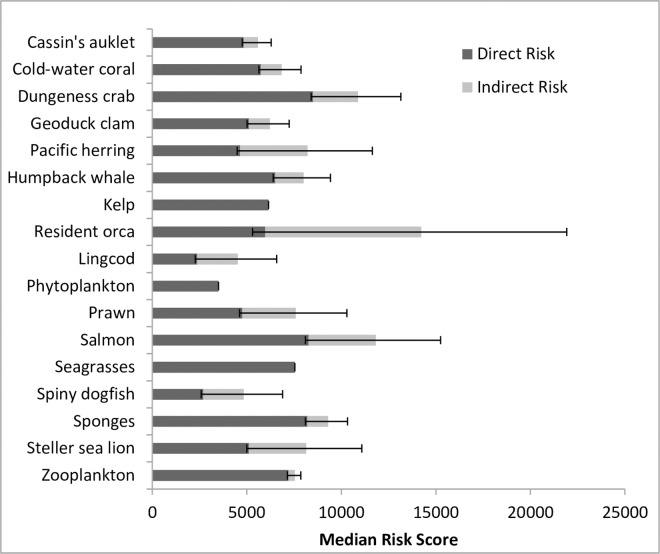
Direct (dark) and indirect (light) median cumulative risk (10/90^th^ Quantile Error bars) to each of the 17 ecosystem components evaluated.

The ecosystem components with the highest comprehensive risk were resident orca, salmon and Dungeness crab (Fig 2). The highest direct risk scores were that for Dungeness crab, salmon, and sponges. The highest indirect risk, mediated through prey and habitat linkages, was for resident orca and salmon. The biggest change with the addition of indirect risk is that for resident orca, for which comprehensive risk is 138% greater than the direct risk alone (Table 3). This is a result of its relatively high direct risk plus the high risk associated with its primary obligate food source–salmon. Salmon and Dungeness crab—which had the next highest comprehensive risk scores—had comprehensive risks 43% and 28% greater than their direct risks, respectively. Also increasing dramatically due to the inclusion of indirect risks were the comprehensive risk scores for lingcod, spiny dogfish, and Pacific herring (Table 3).

**Table 3:** Median comprehensive risk, direct risk, and indirect risk scores for ecosystem components (listed in order of comprehensive risk scores highest to lowest), percentage increase and the number of prey and habitat inputs used to estimate indirect risk.

Ecosystem component	Comprehensive Risk	Direct	Indirect	% Risk Increase	Supporting Species
Resident Orca	14,226	5,969	8,257	138%	4
Salmon	11,827	8,257	3,570	43%	1
Dungeness crab	10,880	8,508	2,371	28%	5
Sponges	9,303	8,199	1,104	13%	2
Herring	8,215	4,644	3,570	77%	2
Steller sea lion	8,144	5,107	3,037	59%	5
Humpback whale	8,008	6,501	1,507	23%	4
Prawn	7,588	4,758	2,830	59%	1
Zooplankton	7,546	7,196	350	5%	2
Seagrasses	7,542	7,542	0	0%	0
Cold water coral	6,847	5,743	1,104	19%	2
Geoduck clam	6,219	5,116	1,104	22%	2
Kelp	6,145	6,145	0	0%	0
Cassin's auklet	5,598	4,844	754	16%	1
Spiny dogfish	4,838	2,688	2,150	80%	3
Lingcod	4,529	2,379	2,150	90%	3
Phytoplankton	3,503	3,503	0	0%	0

**Fig 3:**
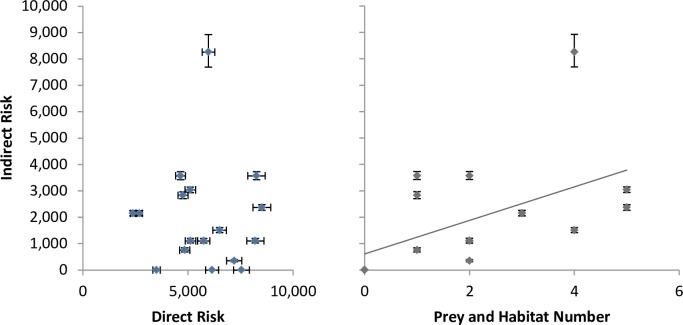
Comparison of median indirect risk scores with direct risk score of each ecosystem component (left panel), and the number of prey and habitats used for a given ecosystem component’s indirect risk score (right panel). X and Y-axis error bars represent 10% and 90% quantiles.

There was no significant relationship between direct and indirect risk scores (Spearman rank, cor = -0.70, df = 16, p = 0.789; Fig 3A). Orcas had moderate direct risk scores, but far greater indirect risk scores than other ecosystem components. In all other species, direct risk scores were greater than indirect. There was a significant correlation between the number of prey and habitat inputs and the indirect risk score (Spearman rank, cor = 0.59, df = 16, p = 0.012; Fig 3B). This relationship appears to be strengthened by resident orca, which has an indirect risk score far above other species.

## Discussion

The incorporation of indirect effects through trophic links and habitat provisioning provides a more comprehensive evaluation of risk to ecosystem components. The comprehensive risk method we present considers potential indirect effects that may alter which species are high priorities for management, improve how species are prioritized within management plans and identify which activities should be regulated to protect at-risk species and the prey and habitats upon which they depend. Our results demonstrate that incorporating food web and habitat relationships into estimates of cumulative risk to ecosystem components changes the risk scores and the relative ranking of ecosystem components. For example, whereas salmon and Dungeness crab are at relatively high risk with or without indirect risks, Pacific herring and Steller sea lion are at relatively low direct risk but at relatively high comprehensive risk. These changes reflect concerns around Pacific herring population levels, climate and overfishing currently being highlighted on the Pacific coast of North America [27]. Direct and indirect risks are relatively independent as we found no relationship between an ecosystem component’s direct risk score and its indirect risk score.

This comprehensive risk framework highlights the importance of activities that might be overlooked without the consideration of indirect risk: activities that threaten prey species or habitats. Resident orca, salmon, Steller sea lion, and humpback whale have legal at risk designations [28–31]. With the exception of humpback whale, which was recently down-graded to Special Concern, all these at-risk species were better represented as high-risk in the comprehensive risk assessment method. Humpback whales remained moderately at risk regardless of risk assessment method. Humpback whales feed low in the food chain and therefore the risk score reflects less accumulation of indirect risk from prey. The historical threats to humpback whale recovery were direct and have largely been removed with the ban of whaling [32]. Ignoring indirect risk can lead to misrepresentation of at-risk species in commonly conducted risk assessments.

The amount of indirect risk is a function of the number of species on which an ecosystem component preys, the substitutability of those prey to the predator, and the risk faced by those prey. By our analyses, indirect risk scores of generalist ecosystem components are correlated with the number of prey and habitat species on which each depends. Thus, by design, by incorporating indirect risk (predation effects), this framework assesses greater risk on those species higher in the food web. Top predators—depending on many prey, which in turn, depend on many prey species.—can be threatened by risks to any of these other species. However, prey switching may buffer some species from risk resulting from one or more of their prey [33]; thus our framework may overestimate risk for some of these species.

Furthermore, ecosystem components with high risk scores do not necessarily have high numbers of prey species. We found an important exception to the relationship between indirect risk and the combined number of prey and habitat linkages. Resident orca are known to specialize on specific prey species [34] and therefore would be likely to experience more risk from its obligate prey species (we specified 100% risk); accordingly, the indirect risk score was much greater than for other generalist species, despite its single prey source. Risk management plans that only include direct risk would be ineffective for species like resident orca, where indirect risk can be much higher than direct risk.

Additionally, evaluating risk through its ecological connections allows management to weigh how individual pathways influence risk. Species of concern, either from a conservation or resource stand-point, can be connected to other high or low risk ecosystem components and these nodes can become important if viewed together with current and potential future human impacts [35]. Ecosystem components connected to others with higher risk levels and the strength of these connections (obligate vs. equivalent) can be used to prescribe appropriate levels of management caution and prevent unexpected resource declines or collapses.

### Limitations and Uncertainties

Cumulative risk scores are highly dependent on the quality and detail of data regarding the linkages between predator and their choice of prey species, the predator’s ability to switch prey species (generalist vs. specialist), and species’ needs for specific habitats. Our study used a 10% risk-transfer relationship, where 10% of risk from a prey species is included as indirect risk to the predatory component, based on simple energy-transfer theory [26]. This 10% risk-transfer assumption may not appropriately represent the incomplete dependency of one species on another, due to substitutability with other prey sources, incomplete spatial overlap between the predator and prey, and context dependent relationships that vary across space and over time. For example, predator species that consume a variety of prey may nevertheless be largely dependent upon one or more species at particular times or life history stages. Calculating substitutability of prey species and spatial overlap is very challenging, so here we used a simple assumption, that risk is diminished an order of magnitude in the transfer. In future cumulative risk assessments, other transfer relationships could be used when there is specific evidence of relative prey dependence and/or habitat utilization. For example, gut content or stable isotope analysis has been used to estimate relative prey importance in marine mammals [34], [36]. Whereas uncertainty is explicitly incorporated in the risk scoring process, we have not incorporated uncertainty regarding prey choice or habitat use. Incorporating multiple potential food web linkages and conducting sensitivity analyses on the structure of the food web for risk scoring could overcome this limitation.

The incorporation of uncertainty scoring directly into the qualitative risk scoring is an advance over qualitative risk scoring methodology as it allows a more complete characterization of risk. Providing the end users and managers with error bars representing uncertainty in the scoring process allows them to visualize the data gaps and assign resources to address them when possible. Uncertainty in the exposure variables can be more easily addressed with additional research or data sharing and allows refinement of resource allocation where overlapping human activities and stressors are a concern. This method of uncertainty incorporation also allows risk assessment in data poor situations and produces risk estimates that can undergo sensitivity analysis to test various scenarios. Use of these methods therefore supports the precautionary approach in ecosystem-based management.

In these types of semi-quantitative risk assessment methods, there is uncertainty and bias introduced by assigning scores to exposure and consequence factors. For example, by multiplying exposure and consequence, the overall risk score is influenced by the factor with higher overall scores. Furthermore, we squared the consequence term in order to balance the influence of these two factors. While this reduces one kind of bias, it inflates the non-linearity of the consequence term, biasing the influence of higher consequence scores on overall risk, a precautionary decision. However, the initial scores themselves are already non-linear, in the sense that—from the ecosystem component’s perspective, as measured by biomass lost, or other such metrics—a score of 3 is more than 50% worse than a score of 2, a score of 5 is more than 25% worse than a score of 4, etc. Further, having an unequal number of variables on either side of the equation affects uncertainty. There are three exposure variables with associated uncertainty and a single variable for consequence, which means that uncertainty can be inflated for exposure. Thus, careful interpretation of the differences in the risk values and the associated uncertainty is warranted.

The risk scores shown here should not be considered predictions of impact. Our risk scores do not account for system thresholds, by which non-linear dynamics can yield long-term impacts from short-term exposures (as but one example). Similarly, because sensitivity of the ecosystem component is evaluated at the scale of individual stressors, the method does not capture contexts in which a slow-maturing, low fecundity species might be vulnerable to population crashes in response to a combination of stressors [37]. Further advances in risk assessment could also account for sensitivity at the scale of each ecosystem component, not just at the scale of individual stressors facing each ecosystem component.

As in previous cumulative effects analyses, our method for estimating cumulative effects currently assumes risk to be additive, though the interaction of various stressors is commonly synergistic, antagonistic or multiplicative [38–40]. There is little known about the mechanisms behind interaction of stressors, and additional study is required to investigate the nature of these relationships using both ecological experimentation and modeling. Even with additional research, the results are likely to be specific to the study area, scale and species and may not be easily generalizable.

Incorporating bottom-up effects of food webs in our comprehensive cumulative risk framework is an advance on commonly used, direct risk frameworks. We did not consider top-down effects [41] or competitive interactions [17], [42–43], which also have the potential to alter risk to these species. Top-down effects are common in marine food webs and have been demonstrated as controlling the structure of marine communities. For example, the presence of sea otters, a higher level marine predator, alters the structure of kelp forest and seagrass ecosystems through their impact on prey populations [44–46]. Competitive interactions can mediate or alter the effects of stressors, either by direct impact on a competitive interdependent species or by altering the nature of the interaction between species [17]. Where information is available about the nature of inter-specific interactions and the vulnerability of each ecosystem component to these system-wide changes, this information could be used to modify the risk assessment framework to better account for the complexity of marine food webs. The complexity of marine ecosystems and their inherent variability across spatial and temporal scales makes estimating the cumulative risk from multiple stressors a difficult and challenging task. Risk assessments must make use of appropriate spatial and temporal scales in order to produce meaningful results and add additional layers of ecological complexity where data exists.

### Conclusions

Risk assessments are a commonly used tool for prioritization of limited management resources and may be used to prioritize species for conservation action or human activities for mitigation of impacts. We showed that including indirect risk in comprehensive risk assessments may improve the evaluation of species at-risk, by incorporating risk mediated through trophic and habitat linkages. The comprehensive cumulative risk framework we propose is easily scalable and can be modified to include new information about top-down or bottom-up ecosystem effects. As this comprehensive risk method is a relatively simple modification of a commonly used risk framework, managers and decision-makers can easily incorporate it into ecosystem-based management, improving our understanding of risk from complex cumulative effects of human activities on marine resources and the ecosystem services they provide.

## Supporting Information

S1 File Qualitative Risk Assessment Scoring. (DOCX)

S2 File R Code for Calculating Cumulative Risk. (DOCX)

S3 File Qualitative Risk Assessment Supplementary Data. (XLSX)
