# Supplementary material for: Supporting Risk Assessment: Accounting for Indirect Risk to Ecosystem Components
Source: PLoS One. 2016 Sep 15;11(9):e0162932. doi: 10.1371/journal.pone.0162932 (PMC5024992; doi:10.1371/journal.pone.0162932)
Supplement: S1 File — (DOCX) [file pone.0162932.s001.docx]

# Supporting Information

## S1 - Qualitative Risk Assessment Scoring

### Scoring exposure and consequence

*Temporal Scale (TS)* refers to the frequency of the event, rather than its duration. Consideration was given to how often the stressor occurs, rather than how long the effect is felt by the ecosystem component (which in practice was included in the *Consequence_ij_* scoring). Scoring is described in S1 Table 1A.

*Spatial Scale* *(SS)* is the scale or spatial extent of the impact from the stressor. For example, under sedimentation from trawl fisheries, consideration was given to how far sediment is carried from the site of the trawl. Scoring for the dive fishery considered the size of the footprint of habitat disturbance from a single dive. Scoring is described in S1 Table 1B.

*Load (L)* is a measure of the density and persistence of the stressor. Depending on the stressor or activity in question, Load can refer to effort, density, amount of an activity, or the amount or strength of a stressor (e.g. quantity or concentration of a pollutant or harmful species, rate of change for climate change) across the entire study area (in this case, the Pacific North Coast Integrated Management Area, PNCIMA). For example, load for finfish aquaculture evaluates how many finfish farms are there in PNCIMA and how often and how much area is covered by finfish farms. Scoring described in S1Table 1C.

*Consequence_ij_* is the impact of the stressor on the individual ecosystem component and therefore must be scored for each ecosystem component -stressor combination. It is scored from 1 to 6 ranging from negligible to intolerable consequence and indicates the impact of the stressor on the individual ecosystem component, as described in S1 Table 1D. *Consequence_ij_* scoring is based on the subcomponent (population size, geographic range, behaviour, etc) but most commonly *Consequence_ij_* was scored on the population size or geographic range subcomponent. If information was available about more than a single subcomponent, the most sensitive subcomponent was used to assign the score. In choosing the most sensitive subcomponent, consideration should be given to the subcomponent most important for long-term persistence and/or the subcomponent that is the most sensitive to the stressor being scored. Uncertainty was also included for the *Consequence_ij_* score; see S1 Table 2 for uncertainty categories and scores.

S1 Table 1. Scoring of *Exposure_i_* variables: a) Spatial Scale, b) Temporal Scale, c) Load and d) *Consequence_ij_*

| **(a) *Temporal Frequency Scale*** | | |
| --- | --- | --- |
| **Score** | **Effect** | **Definition** |
| 1 | Rare | Every several years – Decadal |
| 2 | Relatively Often | Quarterly – Annually |
| 3 | Frequent | Weekly – Monthly |
| 4 | Continuous | Daily occurrences or continuous |

| **(b) *Spatial Scale*** | | |
| --- | --- | --- |
| **Score** | **Effect** | **Definition** |
| 1 | Few restricted locations | 1-10 kilometres |
| 2 | Localized | 10-100 kilometres |
| 3 | Widespread | >100 kilometres |

| **(c) *Load – Density/Persistence*** | | |
| --- | --- | --- |
| **Score** | **Effect** | **Definition** |
| 1 | Low | Low density and low persistence |
| 2 | Moderate | High density or persistence |
| 3 | High | High density and persistence |

| **(d) *Consequence*** | | |
| --- | --- | --- |
| **Score** | **Effect** | **Definition** |
| 1 | Negligible | Negligible impact on population/habitat/community |
| 2 | Minor | Minimal impact on population/habitat/ community structure or dynamics |
| 3 | Moderate | Maximum impact that still meets an objective (e.g. sustainable level of impact such as a full exploitation rate for a target species; maintaining levels of critical habitat) |
| 4 | Major | Wider and longer term impacts (e.g. long-term decline in CPUE) |
| 5 | Severe | Very serious impacts occurring, with a relatively long time period likely to be needed to restore to an acceptable level (e.g. serious decline in spawning biomass limiting population increase) |
| 6 | Intolerable | Widespread and permanent/irreversible damage or loss will occur – unlikely to ever be fixed (e.g. local extinction) |

S1 Table 2. Scoring definition of the uncertainty of risk scores.

| ***Uncertainty*** | | |
| --- | --- | --- |
| **Score** | **Literature** | **Definition** |
| 1 | Extensive | Extensive scientific information; peer-reviewed information; data specific to the location; supported by long-term datasets (10 years or more) |
| 2 | Substantial | Substantial scientific information; non-peer-reviewed information; data specific to the region; supported by recent data (within the last 10 years) or research |
| 3 | Moderate | Moderate level of information; data from comparable regions or older data (more than 10 years) from the area of interest |
| 4 | Limited | Limited information; expert opinion based on observational information or circumstantial evidence |
| 5 | Little to None | Little or no information; expert opinion based on general knowledge |

### Scoring Uncertainty

An “uncertainty incorporation” exercise was completed to include the uncertainty of the qualitative risk scoring in the final and cumulative risk scores. Each risk variable (*Temporal Scale, Spatial Scale, Load* and *Consequence*) was assigned as the mean of a normal distribution with standard deviation set according to the level of uncertainty assigned (S1 Table 2). The distribution was bounded by the minimum and maximum scores for each risk variable so that the scores could not be higher or lower than the variable’s scale (*e.g.*, the intensity score cannot be lower than 1 or higher than 3). The score of each risk variable was then randomly sampled from this distribution with 1000 replicates. The final risk score for each ecosystem component -stressor relationship was a product of the four risk variable arrays (*Risk* = *SS* x *TS* x *L* x *C*^2^), where the first score generated from each variable array is multiplied across all four risk variables, followed by the second, and so on for all 1000 replicates and resulting in a final risk array of 1000 scores. The mean and 10% and 90% quantiles from this final array of the overall risk to each ecosystem component -stressor relationship was reported.

### Sample calculation

For the sample data presented below for the impact of the stressor sea level risk on the ecosystem component, the risk would be estimated as

| **VEC** | **Sector** | **Activity** | **Stressor** | **Load** | **Temporal Scale** | **Spatial Scale** | **Consequence** |
| --- | --- | --- | --- | --- | --- | --- | --- |
| Cassin's Auklet | Long Term | Climate Change | Sea level rise | 1 | 1 | 3 | 4 |

*Risk* = *SS* x *TS* x *L* x *C*^2^

*Risk = 1 x 1 x 3 x 4^2^= 48*

However, with Monte Carlo simulation applied to each variable, the uncertainty value for each variable would be utilized as described in the Scoring Uncertainty section above. The arrays for each variable would be combined into a resultant array of the final cumulative estimate of risk. For the current example, the risk to Cassin’s auklet from sea level rise was estimated to be 63.7 (38.5 – 115.8).
