# Supplementary material for: Supporting Risk Assessment: Accounting for Indirect Risk to Ecosystem Components
Source: PLoS One. 2016 Sep 15;11(9):e0162932. doi: 10.1371/journal.pone.0162932 (PMC5024992; doi:10.1371/journal.pone.0162932)
Supplement: S2 File — (DOCX) [file pone.0162932.s002.docx]

# Supporting Information

## S2 - R Code for Calculating Cumulative Risk

#VEC Risk Pathway

#Developed for use with R by Gerald Singh and Megan Mach

#June 13, 2016

library(car)

#Open file that contains columns of VEC, activity, stressor, Load Score, Temporal Scale Score, Spatial Scale Score and Consequence Risk Scores - Risk Table 17VECs_121217_rformat.csv

imp <- read.csv(file.choose(), stringsAsFactors = FALSE, strip.white = TRUE)

#Label the exposure and consequence variables, assign the columns in the datasheet that contain these data to these labels

imp1<-imp

Load<-as.numeric(imp1$Load)

Temp<-as.numeric(imp1$Temporal.Scale)

Spa<-as.numeric(imp1$Spatial.Scale)

Con<-as.numeric(imp1$Consequence)

#Define uncertainty bounds, where an uncertainty of 1 is assigned a standard deviation of 0.2 while uncertainty of 5 is assigned a standard deviation of 1

unc1<-0.2

unc2<-0.4

unc3<-0.6

unc4<-0.8

unc5<-1

#Apply uncertainty bounds based on code, these are on a scale between 1 and 5

imp1$loadunc<-

ifelse(imp1$U.Load==1,unc1,

ifelse(imp1$U.Load==2,unc2,

ifelse(imp1$U.Load==3,unc3,

ifelse(imp1$U.Load==4,unc4,

ifelse(imp1$U.Load==5,unc5,0)))))#uncertainty around Load

imp1$tempunc<-

ifelse(imp1$U.Temporal==1,unc1,

ifelse(imp1$U.Temporal==2,unc2,

ifelse(imp1$U.Temporal==3,unc3,

ifelse(imp1$U.Temporal==4,unc4,

ifelse(imp1$U.Temporal==5,unc5,0)))))#uncertainty around Temporal

imp1$spacunc<-

ifelse(imp1$U.Spatial.scale==1,unc1,

ifelse(imp1$U.Spatial.scale==2,unc2,

ifelse(imp1$U.Spatial.scale==3,unc3,

ifelse(imp1$U.Spatial.scale==4,unc4,

ifelse(imp1$U.Spatial.scale==5,unc5,0)))))#uncertainty around Spatial

imp1$concunc<-

ifelse(imp1$U.consequence==1,unc1,

ifelse(imp1$U.consequence==2,unc2,

ifelse(imp1$U.consequence==3,unc3,

ifelse(imp1$U.consequence==4,unc4,

ifelse(imp1$U.consequence==5,unc5,0)))))#uncertainty around Consequence

#Random selection of risk score based on normal distribution curve as assigned by the uncertainty score (and assigned standard deviation), replicated 1000 times

spac<-paste(imp1[,1],",",imp1[,3],",",imp1[,4]) #assign the columns that will be the label for each row, in our data sheet column 1 is VEC, column 3 is Activity, and column 4 is Stressor. Comma’s were added for easy splitting of text to columns in Excel for viewing and sorting results.

randrisk<-array(NA,dim=c(nrow(imp1),4,1000))

for(k in 1:1000){

for(i in 1:nrow(imp1)) {

randrisk[i,1,k]<-Load[i]+(rnorm(1,0,imp1$loadunc[i]))

randrisk[i,2,k]<-Temp[i]+(rnorm(1,0,imp1$tempunc[i]))

randrisk[i,3,k]<-Spa[i]+(rnorm(1,0,imp1$spacunc[i]))

randrisk[i,4,k]<-Con[i]+(rnorm(1,0,imp1$concunc[i]))

}

rownames(randrisk)<-spac

colnames(randrisk)<-c("Load","Temp","Spa","Con")

}

#Sets the upper and lower bounds so risk scores do not go above or below the range of the variables being sampled

randrisk[,1,]<-recode(randrisk[,1,],"lo:1=1");randrisk[,1,]<-recode(randrisk[,1,],"3:hi=3")

randrisk[,2,]<-recode(randrisk[,2,],"lo:1=1");randrisk[,2,]<-recode(randrisk[,2,],"4:hi=4")

randrisk[,3,]<-recode(randrisk[,3,],"lo:1=1");randrisk[,3,]<-recode(randrisk[,3,],"3:hi=3")

randrisk[,4,]<-recode(randrisk[,4,],"lo:1=1");randrisk[,4,]<-recode(randrisk[,4,],"6:hi=6")

#hist(randrisk[1,4,],breaks=50)

#### Producing Risk Scores ####

#### Raw Scores####

#Produce summary statistics on the various risk scores giving median and error (the 10 and 90% quantiles) for every VEC and stressor interaction across all 1000 runs

stat<-array(NA,dim=c(nrow(imp1),12))

for(i in 1:nrow(imp1)) {

stat[i,1]<-median(randrisk[i,1,1:k],na.rm=T)

stat[i,2]<-quantile(randrisk[i,1,1:k],probs=c(0.1),names=F,na.rm=T)

stat[i,3]<-quantile(randrisk[i,1,1:k],probs=c(0.9),names=F,na.rm=T)

stat[i,4]<-median(randrisk[i,2,1:k],na.rm=T)

stat[i,5]<-quantile(randrisk[i,2,1:k],probs=c(0.1),names=F,na.rm=T)

stat[i,6]<-quantile(randrisk[i,2,1:k],probs=c(0.9),names=F,na.rm=T)

stat[i,7]<-median(randrisk[i,3,1:k],na.rm=T)

stat[i,8]<-quantile(randrisk[i,3,1:k],probs=c(0.1),names=F,na.rm=T)

stat[i,9]<-quantile(randrisk[i,3,1:k],probs=c(0.9),names=F,na.rm=T)

stat[i,10]<-median(randrisk[i,4,1:k],na.rm=T)

stat[i,11]<-quantile(randrisk[i,4,1:k],probs=c(0.1),names=F,na.rm=T)

stat[i,12]<-quantile(randrisk[i,4,1:k],probs=c(0.9),names=F,na.rm=T)

rownames(stat)<-spac

colnames(stat)<-c("LoadMed","10%Q","90%Q","TempMed","10%Q","90%Q","SpaMed","10%Q","90%Q","ConMed","10%Q","90%Q")

}

####Produce Raw Exposure and Consequence Scores across 1000 iterations ###

expcon<-array(NA,dim=c(nrow(imp1),2,1000))

for(i in 1:nrow(imp1)) {

for(k in 1:1000){

expcon[i,1,k]<-randrisk[i,1,k]*randrisk[i,2,k]*randrisk[i,3,k]

expcon[i,2,k]<-randrisk[i,4,k]^2

rownames(expcon)<-spac

colnames(expcon)<-c("Exposure","Consequence")

}

}

###Produce Raw Risk Scores across 1000 iterations###

risk<-array(NA,dim=c(nrow(imp1),1,1000))

for(i in 1:nrow(imp1)) {

for(k in 1:1000){

risk[i,1,k]<-randrisk[i,1,k]*randrisk[i,2,k]*randrisk[i,3,k]*randrisk[i,4,k]^2

rownames(risk)<-spac

colnames(risk)<-"Risk"

}

}

#hist(risk[1,1,],breaks=50)

####Raw Summary Stats for exposure and consequence for every VECxActivity/Stressor combination###

statexpcon<-array(NA,dim=c(nrow(imp1),6))

for(i in 1:nrow(imp1)) {

statexpcon[i,1]<-median(expcon[i,1,1:k],na.rm=T)

statexpcon[i,2]<-quantile(expcon[i,1,1:k],probs=c(0.1),names=F,na.rm=T)

statexpcon[i,3]<-quantile(expcon[i,1,1:k],probs=c(0.9),names=F,na.rm=T)

statexpcon[i,4]<-median(expcon[i,2,1:k],na.rm=T)

statexpcon[i,5]<-quantile(expcon[i,2,1:k],probs=c(0.1),names=F,na.rm=T)

statexpcon[i,6]<-quantile(expcon[i,2,1:k],probs=c(0.9),names=F,na.rm=T)

rownames(statexpcon)<-spac

colnames(statexpcon)<-c("ExpMed","10% Quantile","90% Quantile","ConMed","10% Quantile","90% Quantile")

}

###Record Exposure and Consequence summary stats into .csv files for every VECxActivity/Stressor combination###

write.table(statexpcon,file="RiskScoresExpConCalc.csv",col.names=NA,sep=",") #Summary table for every Exposure and Consequence Score

#write.table(logstatexpcon,file="RiskScoresExpConCalcTrans.csv",col.names=NA,sep=",") #Summary table for every Exposure and Consequence Score.

#Summary Stats for Raw Risk Scores###

statrisk<-array(NA,dim=c(nrow(imp1),3))

for(i in 1:nrow(imp1)) {

statrisk[i,1]<-median(risk[i,1,1:k],na.rm=T)

statrisk[i,2]<-quantile(risk[i,1,1:k],probs=c(0.1),names=F,na.rm=T)

statrisk[i,3]<-quantile(risk[i,1,1:k],probs=c(0.9),names=F,na.rm=T)

rownames(statrisk)<-spac

colnames(statrisk)<-c("RiskMed","10% Quantile","90% Quantile")

}

###Record output into .csv files for every VECxActivity combination###

write.table(stat,file="RiskScoresExpCon.csv",col.names=NA,sep=",") #Summary table for every Exposure and Consequence Score

write.table(statrisk,file="RiskScoresRaw.csv",col.names=NA,sep=",") #Summary table for combined raw risk score for every VEC x activity combination

####Look at Cumulative Risk for Raw Scores#####

species<-imp1[,1]

as.list(species)

agrisk<-aggregate(risk,list(species),sum,na.rm=T)

agriska<-as.data.frame(t(agrisk[,-1])) ##Creates a dataframe with all cumulative risk scores across 1000 replications

colnames(agriska)<-agrisk[,1]

##Summary Stats for Cumulative Risk Scores

statagrisk<-array(NA,dim=c(length(agrisk[,1]),3))

for(i in 1:length(agrisk[,1])) {

statagrisk[i,1]<-median(agriska[1:length(agriska[,1]),i])

statagrisk[i,2]<-quantile(agriska[1:length(agriska[,1]),i],probs=c(0.1),names=F)

statagrisk[i,3]<-quantile(agriska[1:length(agriska[,1]),i],probs=c(0.9),names=F)

rownames(statagrisk)<-agrisk[,1]

colnames(statagrisk)<-c("DirectRiskMed","10%Q","90%Q")

}

write.table(statagrisk,file="DirectRiskVEC.csv",col.names=NA,sep=",") #Summary table for Direct risk to VECs

################Look at Risk to VECS - Direct, Indirect and Total########################

#############Original Scale####################

PhytoD <- agriska$Phytoplankton

ZooD <- agriska$Zooplankton

SeagrassD <- agriska$Seagrasses

KelpD <- agriska$Kelp

SpongeD <- agriska$Sponges

CoralD <- agriska[,2]

GeoduckD <- agriska[,4]

DungCrabD <- agriska[,3]

PrawnD <- agriska$Prawn

HerringD <- agriska$Herring

LingcodD <- agriska$Lingcod

SalmonD <- agriska[,12]

DogfishD <- agriska[,14]

HumpbackD <- agriska[,6]

OrcaD <- agriska[,8]

SeaLionD <- agriska[,16]

AukletD <- agriska[,1]

######Lower Trophic Level Groupings for Risk Pathway:######

PhytoR <-vector(length=1000)

ZooR<-vector(length=1000)

SeagrassR<-vector(length=1000)

KelpR<-vector(length=1000)

PelagicFishR<-vector(length=1000)

LowMobileInvertR<-vector(length=1000)

MobileBenthicInvertR<-vector(length=1000)

MobilePelagicInvertR<-vector(length=1000)

BenthicFishR<-vector(length=1000)

AnadroFishR<-vector(length=1000)

for(i in 1:1000){

PhytoR[i] = PhytoD[i]

ZooR[i]<-ZooD[i] + (.1*PhytoR[i])

SeagrassR[i] = SeagrassD[i]

KelpR[i] = KelpD[i]

}

for(i in 1:1000){

LowMobileInvertR[i] = GeoduckD[i] + (.1*ZooR[i]) + (.1 * PhytoR[i])

}

for(i in 1:1000){

MobileBenthicInvertR[i] = DungCrabD[i] + (.1*LowMobileInvertR[i]) + (.1*MobileBenthicInvertR[i]) + (.1*SeagrassD[i])

MobilePelagicInvertR[i] = PrawnD[i] + (.1*MobilePelagicInvertR[i])+ (.1*LowMobileInvertR[i]) + (.1*ZooR[i])+ (.1*SeagrassD[i])

PelagicFishR[i] = HerringD[i] + (.1*PelagicFishR[i]) + (.1*MobilePelagicInvertR[i]) + (.1*ZooR[i]) + (.1*SeagrassD[i]) + (.1*KelpD[i])

BenthicFishR[i] = LingcodD[i] + (.1*BenthicFishR[i]) + (.1*PelagicFishR[i]) + (.1*MobileBenthicInvertR[i])

AnadroFishR[i] = SalmonD[i] + (.1*PelagicFishR[i]) + (.1*MobilePelagicInvertR[i]) + (.1*ZooR[i])+ (.1*SeagrassD[i]) + (.1*KelpD[i])

}

#####VEC Risk Pathways: (Direct and Indirect)######

Phyto = PhytoD

Zoo = ZooR

Seagrass = SeagrassD

Kelp = KelpD

Sponge<-vector(length=1000)

Coral<-vector(length=1000)

Geoduck<-vector(length=1000)

DungCrab<-vector(length=1000)

Prawn<-vector(length=1000)

Herring<-vector(length=1000)

Lingcod<-vector(length=1000)

Salmon<-vector(length=1000)

Dogfish<-vector(length=1000)

Humpback<-vector(length=1000)

Orca<-vector(length=1000)

SeaLion<-vector(length=1000)

Auklet<-vector(length=1000)

for(i in 1:1000){

Sponge[i] = SpongeD[i] + (.1*ZooR[i]) + (.1 * PhytoR[i])

Coral[i] = CoralD[i] + (.1*ZooR[i]) + (.1 * PhytoR[i])

Geoduck[i] = GeoduckD[i] + (.1*ZooR[i]) + (.1 * PhytoR[i])

DungCrab[i] = DungCrabD[i] + (.1*LowMobileInvertR[i]) + (.1*MobileBenthicInvertR[i]) + (.1*SeagrassD[i])

Prawn[i] = PrawnD[i] + (.1*MobilePelagicInvertR[i]) + (.1*LowMobileInvertR[i]) + (.1*ZooR[i]) + (.1*SeagrassD[i])

Herring[i] = HerringD[i] + (.1*PelagicFishR[i]) + (.1*MobilePelagicInvertR[i]) + (.1*ZooR[i]) + (.1*SeagrassD[i]) + (.1*KelpD[i])

Lingcod[i] = LingcodD[i] + (.1*BenthicFishR[i]) + (.1*PelagicFishR[i]) + (.1*MobileBenthicInvertR[i])

Salmon[i] = SalmonD[i] + (.1*PelagicFishR[i]) + (.1*MobilePelagicInvertR[i]) + (.1*ZooR[i])+ (.1*SeagrassD[i]) + (.1*KelpD[i])

Dogfish[i] = DogfishD[i] + (.1*BenthicFishR[i]) + (.1*PelagicFishR[i]) + (.1*MobileBenthicInvertR[i])

Humpback[i] = HumpbackD[i] + (.1*PelagicFishR[i]) + (.1*ZooR[i])

Orca[i] = OrcaD[i] + (SalmonD[i]) # we use 100% risk to salmon because resident orca’s only eat salmon, thus high risk

SeaLion[i] = SeaLionD[i] + (.1*BenthicFishR[i]) + (.1*PelagicFishR[i]) + (.1*AnadroFishR[i]) + (.1*MobilePelagicInvertR[i])

Auklet[i] = AukletD[i] + (.1*ZooR[i])

}

####Look at Food Web impacts on Risk scores for VECS

VECriskmed<-rbind(median(Phyto),

median(Zoo),

median(Seagrass),

median(Kelp),

median(Sponge),

median(Coral),

median(Geoduck),

median(DungCrab),

median(Prawn),

median(Herring),

median(Lingcod),

median(Salmon),

median(Dogfish),

median(Humpback),

median(Orca),

median(SeaLion),

median(Auklet))

qVECrisk<-rbind(quantile(Phyto,probs=c(0.1,0.9),names=F),

quantile(Zoo,probs=c(0.1,0.9),names=F),

quantile(Seagrass,probs=c(0.1,0.9),names=F),

quantile(Kelp,probs=c(0.1,0.9),names=F),

quantile(Sponge,probs=c(0.1,0.9),names=F),

quantile(Coral,probs=c(0.1,0.9),names=F),

quantile(Geoduck,probs=c(0.1,0.9),names=F),

quantile(DungCrab,probs=c(0.1,0.9),names=F),

quantile(Prawn,probs=c(0.1,0.9),names=F),

quantile(Herring,probs=c(0.1,0.9),names=F),

quantile(Lingcod,probs=c(0.1,0.9),names=F),

quantile(Salmon,probs=c(0.1,0.9),names=F),

quantile(Dogfish,probs=c(0.1,0.9),names=F),

quantile(Humpback,probs=c(0.1,0.9),names=F),

quantile(Orca,probs=c(0.1,0.9),names=F),

quantile(SeaLion,probs=c(0.1,0.9),names=F),

quantile(Auklet,probs=c(0.1,0.9),names=F))

VEC<-list("Phytoplankton",

"Zooplankton" ,

"Seagrass" ,

"Kelp",

"Sponge" ,

"Coral" ,

"Geoduck" ,

"DungCrab" ,

"Prawn" ,

"Herring" ,

"Lingcod" ,

"Salmon" ,

"Dogfish",

"Humpback" ,

"Orca",

"SeaLion",

"Auklet")

VECrisk<-cbind(VECriskmed,qVECrisk)

rownames(VECrisk)<-VEC

colnames(VECrisk)<-c("VECTotalriskmed","10% quantile","90% quantile")

write.table(VECrisk,file="CumulativeRiskVEC.csv",col.names=NA,sep=",") #Summary table for aggregate risk (direct and indirect) and error quantiles to Ecosystem Components

#################################

#Calculate Indirect Risk only ###

#################################

for(i in 1:1000){

PhytoI = 0

SeagrassI = 0

KelpI = 0

}

ZooI<-vector(length=1000)

SpongeI<-vector(length=1000)

CoralI<-vector(length=1000)

GeoduckI<-vector(length=1000)

DungCrabI<-vector(length=1000)

PrawnI<-vector(length=1000)

HerringI<-vector(length=1000)

LingcodI<-vector(length=1000)

SalmonI<-vector(length=1000)

DogfishI<-vector(length=1000)

HumpbackI<-vector(length=1000)

OrcaI<-vector(length=1000)

SeaLionI<-vector(length=1000)

AukletI<-vector(length=1000)

for(i in 1:1000){

ZooI[i] = (.1*PhytoR[i])

SpongeI[i] = (.1*ZooR[i]) + (.1 * PhytoR[i])

CoralI[i] = (.1*ZooR[i]) + (.1 * PhytoR[i])

GeoduckI[i] = (.1*ZooR[i]) + (.1 * PhytoR[i])

DungCrabI[i] = (.1*LowMobileInvertR[i]) + (.1*MobileBenthicInvertR[i]) + (.1*SeagrassD[i])

PrawnI[i] = (.1*MobilePelagicInvertR[i]) + (.1*LowMobileInvertR[i]) + (.1*ZooR[i]) + (.1*SeagrassD[i])

HerringI[i] = (.1*PelagicFishR[i]) + (.1*MobilePelagicInvertR[i]) + (.1*ZooR[i]) + (.1*SeagrassD[i]) + (.1*KelpD[i])

LingcodI[i] = (.1*BenthicFishR[i]) + (.1*PelagicFishR[i]) + (.1*MobileBenthicInvertR[i])

SalmonI[i] = (.1*PelagicFishR[i]) + (.1*MobilePelagicInvertR[i]) + (.1*ZooR[i])+ (.1*SeagrassD[i]) + (.1*KelpD[i])

DogfishI[i] = (.1*BenthicFishR[i]) + (.1*PelagicFishR[i]) + (.1*MobileBenthicInvertR[i])

HumpbackI[i] = (.1*PelagicFishR[i]) + (.1*ZooR[i])

OrcaI[i] = (SalmonD[i]) # we use 100% risk to salmon because resident orca’s only eat salmon, thus high risk

SeaLionI[i] = (.1*BenthicFishR[i]) + (.1*PelagicFishR[i]) + (.1*AnadroFishR[i]) + (.1*MobilePelagicInvertR[i])

AukletI[i] = (.1*ZooR[i])

}

####Look at Food Web impacts on Risk scores for VECS

VECIndirectriskmed<-rbind(median(PhytoI),

median(ZooI),

median(SeagrassI),

median(KelpI),

median(SpongeI),

median(CoralI),

median(GeoduckI),

median(DungCrabI),

median(PrawnI),

median(HerringI),

median(LingcodI),

median(SalmonI),

median(DogfishI),

median(HumpbackI),

median(OrcaI),

median(SeaLionI),

median(AukletI))

qVECIndirectrisk<-rbind(quantile(PhytoI,probs=c(0.1,0.9),names=F),

quantile(ZooI,probs=c(0.1,0.9),names=F),

quantile(SeagrassI,probs=c(0.1,0.9),names=F),

quantile(KelpI,probs=c(0.1,0.9),names=F),

quantile(SpongeI,probs=c(0.1,0.9),names=F),

quantile(CoralI,probs=c(0.1,0.9),names=F),

quantile(GeoduckI,probs=c(0.1,0.9),names=F),

quantile(DungCrabI,probs=c(0.1,0.9),names=F),

quantile(PrawnI,probs=c(0.1,0.9),names=F),

quantile(HerringI,probs=c(0.1,0.9),names=F),

quantile(LingcodI,probs=c(0.1,0.9),names=F),

quantile(SalmonI,probs=c(0.1,0.9),names=F),

quantile(DogfishI,probs=c(0.1,0.9),names=F),

quantile(HumpbackI,probs=c(0.1,0.9),names=F),

quantile(OrcaI,probs=c(0.1,0.9),names=F),

quantile(SeaLionI,probs=c(0.1,0.9),names=F),

quantile(AukletI,probs=c(0.1,0.9),names=F))

VEC<-list("Phytoplankton",

"Zooplankton" ,

"Seagrass" ,

"Kelp",

"Sponge" ,

"Coral" ,

"Geoduck" ,

"DungCrab" ,

"Prawn" ,

"Herring" ,

"Lingcod" ,

"Salmon" ,

"Dogfish",

"Humpback" ,

"Orca",

"SeaLion",

"Auklet")

VECIndirectrisk<-cbind(VECIndirectriskmed,qVECIndirectrisk)

rownames(VECIndirectrisk)<-VEC

colnames(VECIndirectrisk)<-c("VECIndirectriskmed","10% quantile","90% quantile")

write.table(VECIndirectrisk,file="IndirectRiskVEC.csv",col.names=NA,sep=",") #Summary table for indirect risk and error quantiles to Ecosystem Components
